# Effects of Fermentation Modification and Combined Modification with Heat-Moisture Treatment on the Multiscale Structure, Physical and Chemical Properties of Corn Flour and the Quality of Traditional Fermented Corn Noodles

**DOI:** 10.3390/foods13244043

**Published:** 2024-12-14

**Authors:** Chen Mao, Sijia Wu, Ling Zhang, Hong Zhuang

**Affiliations:** 1 College of Food Science and Engineering, Jilin University, Changchun 130062, China; maochen_workmail@163.com (C.M.); zhang_ling@jlu.edu.cn (L.Z.); 2 Shenzhen Institutes of Advanced Technology, Chinese Academy of Sciences, Shenzhen 518055, China; wusijia2017@sina.com

**Keywords:** fermentation, heat-moisture treatment, combined modification, noodles, multi-scale structures

## Abstract

This study investigates the effects of fermentation modification and combined modification with heat-moisture treatment (HMT) on the multiscale structure, physical and chemical properties, and quality of corn flour in the production of traditional fermented corn noodles (TFCNs). The results indicate that after fermentation modification, the starch granule size decreased while the amylopectin proportion increased. Fermentation also enhanced the relative crystallinity and short-range order of the starch, along with an increase in resistant digestion components and ester content in the noodles. After combined modification with HMT, starch granules lost their spherical, intact structure, underwent melting and reorganization, and displayed an increase in particle size. These changes led to a significant improvement in the thermal stability and textural properties of corn flour, resulting in noodles with enhanced cooking quality. Furthermore, the combined modification significantly increased the contents of flavor compounds such as aldehydes, acids, and alcohols in the noodles while reducing olefin and alkane levels, thus contributing to improved flavor development. These findings demonstrate that fermentation modification and combined modification with HMT play a crucial role in enhancing the multiscale structure and physical and chemical properties of corn starch, thereby improving the quality of TFCN.

## 1. Introduction

The enhancement of cereal starch structure and functional properties through various modification methods and its impact on food quality has become a major research focus in the field of food science in recent years. Corn, a member of the Poaceae family, ranks as the third most-produced cereal globally (following wheat and rice), with its yield nearly quintupling over the past 60 years. Corn starch, which constitutes approximately 75% of the corn’s dry matter, is a key determinant of corn starch-based staple food quality due to its physical and chemical properties, including particle size, the amylose-to-amylopectin ratio, crystallinity, and thermal performance [1,2]. As an economical, gluten-free (GF) ingredient, corn starch is ideal for GF foods, contributing to improved texture and flavor. Therefore, corn starch-based foods hold promise as an excellent staple alternative and daily nutritional option for individuals with gluten intolerance.

However, natural cereal starches like corn starch generally have poor processing performance, leading to noodles with insufficient elasticity, structural stability, and anti-regeneration properties, which limits their application in staple food products. Various modification methods, including physical (such as ultrasound, heat-moisture treatment, and dry heat treatment), chemical (such as esterification, etherification, and oxidation), and biological modifications (such as enzyme treatment and fermentation), have been shown to significantly improve starch processing performance, better meeting the diverse needs of producers and consumers [3,4]. Building on this, to overcome the limitations of single modification techniques, combined modification methods have been proposed and categorized by Ashogbon et al. [5] into two main types: homogeneous and heterogeneous combined modifications. Homogeneous combined modifications involve using two or more methods of the same type, including physical/physical (such as HMT/high-pressure combined modification), chemical/chemical (such as acetylation/etherification combined modification), and biological/biological methods (such as α-amylase/pullulanase combined modification). In contrast, heterogeneous combined modifications employ two or more different types of modification methods, including physical/chemical (such as acetylation/HMT combined modification), physical/biological (such as high-frequency ultrasound/α-amylase combined modification), and chemical/biological methods (such as acetylation/α-amylase combination).

It is noteworthy that even before the application of new technologies, some traditional food processing methods have involved starch modification and its combined use, and the modification mechanisms derived from these traditional processes warrant further exploration. Taking traditional fermented corn noodles (TFCN) as an example, TFCN differs from noodles made with blended flour, as it consists solely of fermented corn flour and is gluten-free, making it suitable for individuals on gluten-free diets. The production process includes two key steps: (1) fermenting corn flour with lactic acid bacteria and (2) cooking the fermented corn flour in hot water before forming it into noodles. This process inherently includes both fermentation-induced starch modification and its combined modification with heat-moisture treatment (HMT).

Fermentation modification is a key initial step in the production of TFCN. The enzymes and organic acids secreted by lactic acid bacteria promote the degradation of starch molecules, altering the structure and ratio of amylose and amylopectin. This process can improve the texture, flavor components, and cooking quality of the noodles [6]. Heat-moisture treatment (HMT) is a widely used, safe, and effective method for starch modification. Under conditions of moisture content < 35%, temperature > 90 °C, and treatment time > 15 min, starch undergoes expansion, collapse, degradation, cross-linking, and directional rearrangement. This results in a more stable gel network structure [7], which contributes to producing TFCN with excellent cooking quality, strong retrogradation resistance, and good storage stability.

Although there are related studies on the combined modification of starch, the study of modification technology based on traditional food processing and its combined modification technology is helpful in strengthening the connection between modification technology and food production. In addition, there are no reports on the combined modification of fermentation and HMT, and the effects of combined modification on the multi-scale structure of starch granules, the physical and chemical properties of corn flour, and the quality of TFCN are still unclear. Therefore, this study explored the effects of fermentation modification and its combined modification with HMT on the starch content, micromorphology, ordered structure, physical and chemical properties, and texture characteristics of corn flour, as well as the cooking quality, digestive characteristics, and flavor components of noodles during the production of TFCN. This study aims to clarify the mechanism of the influence of fermentation modification and combined modification with HMT on corn flour during the production of TFCN, fill the research gap of fermentation and HMT combined modification of corn flour and provide a reference for the production of higher quality TFCN.

## 2. Materials and Methods

### 2.1. Materials and Reagents

Fresh corn (Jilin Zhongpinyuan Agricultural Development Co., Ltd., Changchun, China), *Lactobacillus plantarum* (Lyophilized powder, ATCC 8014, Shanghai Shifeng Biotechnology Co., Ltd., Shanghai, China), MRS medium (HB0384, Qingdao Haibo Biotechnology Co., Ltd., Qingdao, China), PBS buffer (BL601A, Beijing Lanjieke Technology Co., Ltd., Beijing, China), and an amylose/amylopectin content detection kit (BC4265, Solarbio Science & Technology Co., Ltd., Beijing, China) were purchased. All chemical reagents were of analytical grade.

### 2.2. Sample Preparation

**Figure 1:**
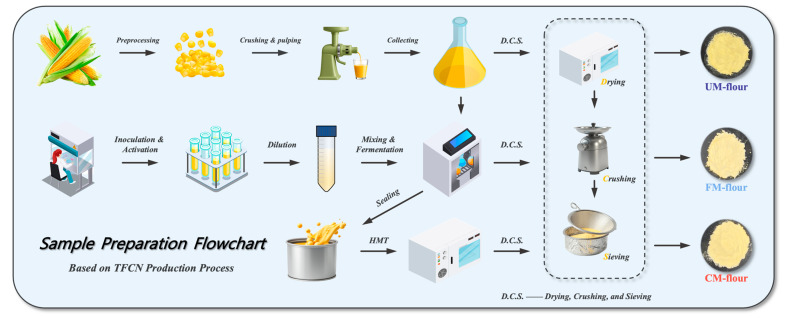
Sample Preparation Flowchart of UM, FM, and CM.

Based on the production process of TFCN, the preparation processes of unmodified corn flour (UM-flour), fermented modified corn flour (FM-flour), and fermented and HMT combined modified corn flour (CM-flour) were designed, as shown in Figure 1.

Preparation of UM-flour: Fresh corn kernels were mixed with an equal weight of distilled water, crushed to obtain corn slurry, and then subjected to the subsequent D.C.S. step to obtain UM-flour.

Preparation of FM-flour: Freeze-dried *Lactobacillus plantarum* powder was added to sterile MRS medium and activated at 37 °C for 48 h, and the bacterial suspension concentration was adjusted to 10^8^ CFU/mL. The batches were transferred to sterile centrifuge tubes, and the culture medium was washed away with PBS buffer and resuspended to obtain the fermentation broth. The fermentation broth was mixed with PBS at a specific volume ratio (the optimal concentration determined by preliminary research is 17% (*w*/*w*)) to obtain the fermentation suspension. It was combined with corn slurry, and the initial pH was adjusted to 7.0 with 1 M NaOH or HCl and incubated at 45 °C for 3 d. It was centrifuged at 5000 rpm for 5 min, the precipitate was collected, washed with an appropriate amount of distilled water, and D.C.S. steps were used to obtain FM-flour.

Preparation of CM-flour: The above-separated precipitate was collected and sealed in a container, the moisture content was adjusted to 30%, non-contact heated at 100 °C for 1 h, cooled, and the D.C.S. step was completed to obtain CM-flour.

Starch extraction: Improved on the basis of Zhang et al. [8]. A total of 100 g of the sample prepared above was mixed with 500 mL of distilled water and soaked for 2 h. Then, it was centrifuged several times at 5000 rpm to remove the supernatant, and the yellow part of the precipitate was scraped off. The residue was washed with distilled water, and the white starch was mixed with ethanol and filtered. After the D.C.S. step, starch was obtained.

D.C.S. (Drying, Crushing, and Sieving): All samples were hot-air dried at 50 °C for 24 h, then crushed at low temperature and passed through a 100-mesh sieve several times. The powder was collected and stored at 4 °C for later use.

### 2.3. Starch Content, Color Characteristics, and Particle Size Distribution

According to the instructions, the content of amylose and amylopectin in the sample was determined using the amylose/amylopectin content detection kit. Specifically, the amylopectin content was calculated by measuring the difference between the total starch content and the amylose content in the samples.

The color characteristics of the samples were measured using a Colorimeter (CR-400, Konica Minolta Sensing Americas Inc., Ramsey, NJ, USA). Before the test, the instrument was calibrated with a white calibration plate. The results were expressed as L* (brightness value), a* (red/green value), and b* (yellow/blue value) in the CIE color space.

The particle size of the samples was measured according to the method of Wang et al. [9] with slight modifications. Prior to measurement, 0.1 g of the sample was suspended in 0.9 mL of deionized water and dispersed in the diffraction cell until the opacity reached 10%, as required by the instrument. The measurement was performed using a laser particle size analyzer (Mastersizer 3000, Malvern Instruments Ltd., Worcester, UK).

### 2.4. Starch Microstructure

The microstructure of starch granules was observed using a Scanning Electron Microscope (SEM, SU8600, Hitachi High-Tech Science, Tokyo, Japan). All samples were freeze-dried and evenly distributed on a sample holder coated with conductive double-sided tape. A layer of palladium alloy and a layer of carbon powder were sputtered onto the samples under vacuum conditions. The operating conditions were an acceleration voltage of 15 kV, a vacuum pressure of 10^−6^ Pa, and magnifications of 1.0 kx and 6.0 kx.

The Maltese cross of starch granules was observed using a polarizing microscope (Leica DM4 P, Leica Microsystems, Wetzlar, Germany).

### 2.5. Solubility, Water Holding Capacity (WHC), and Retrogradation Properties

The solubility, WHC, and retrogradation properties of the sample were determined using the method of Wu et al. [10] with slight modifications. A sample powder with mass M was mixed with 20 mL of deionized water at a 1:10 (*w*/*v*) ratio and placed in a centrifuge tube with mass M0. The mixture was vortexed for 10 min. The mixture was then heated in a water bath at 50, 60, 70, 80, and 90 °C for 30 min, wiped dry, and allowed to cool to room temperature. The mass of the centrifuge tube and its contents was recorded as M_1_. The mixture was then centrifuged at 4000 rpm for 15 min to separate the supernatant. The mass of the centrifuge tube and the remaining material was recorded as M_2_. The separated supernatant was dried in a hot air oven at 80 °C until a constant weight was achieved. The mass of the residue was recorded as M_3_. The solubility was calculated using Formula (1), and the WHC was calculated using Formula (2).

For the retrogradation properties determination, 100 mL of a 1% (*w*/*v*) sample powder suspension in deionized water was prepared, sealed in a cylinder, and heated in a 90 °C water bath for 30 min. After cooling, the sample was transferred to a 100 mL measuring cylinder, the cylinder was sealed, and the precipitation volume (V) was recorded at 0, 2, 4, 6, 8, 10, 12, and 24 h. The retrogradation properties were calculated according to Formula (3).

(1) $$\mathrm{Solubility}\;\left(\%\right)=\frac{M_{3}}{M}\;\times \;100\%$$

(2) $$\mathrm{WHC}\;\left(\%\right)=\frac{M_{2}-M_{0}}{M_{1}-M_{0}}\;\times \;100\%$$

(3) $$\mathrm{Retrogradation}\;\mathrm{Characteristics}\;\left(\%\right)=\frac{100-V}{V}\;\times \;100\%$$

### 2.6. X-Ray Diffraction (XRD)

XRD analysis was performed on the samples using a high-resolution X-ray diffractometer (X’Pert3 MRD, Malvern Panalytical, Almelo, The Netherlands). The powdered samples were placed in the grooves of the scanning disk and compacted for measurement, then scanned under a Cu-target graphite monochromator. The experimental conditions included a voltage of 40 kV, a current of 40 mA, and a scanning rate of 5°/min from 5° to 40° (2θ) at room temperature. Characteristic peak analysis and the calculation of relative crystallinity (RC) were performed using MDI Jade 9.0 software (Material Data, Inc., Livermore, CA, USA). The formula is shown in (4):

(4) $$RC\;(\%)=\frac{A_{c}}{A_{c}+A_{a}}\times 100$$

### 2.7. Fourier Transform Infrared Spectroscopy (FT-IR)

Infrared spectra of the samples were measured using an FT-IR spectrometer (Nicolet iS50, Thermo Fisher Scientific, Waltham, MA, USA). The powder was mixed with KBr, ground into pellets, and then analyzed. The scanning range was from 4000 to 400 cm^−1^, with each sample scanned 32 times at a resolution of 4 cm^−1^. After obtaining the FT-IR data, baseline correction was performed on the collected infrared spectra using Omnic 9.2 software (Thermo Fisher Scientific, Madison, WI, USA), and deconvolution of the infrared spectra in the range of 1200 to 800 cm^−1^ was conducted.

### 2.8. Dynamic Rheological Property

The method of Yang et al. [11] was followed and improved. In brief, the sample powder was weighed and mixed with deionized water to prepare a 10% (*w*/*w*) starch suspension, which was then heated in a 90 °C water bath for 30 min to form a starch paste. The dynamic rheological properties of the starch paste were evaluated at 25 °C using a rheometer equipped with parallel plates, with a strain of 0.5% and an angular frequency range from 0.1 to 100 rad/s. During the measurement, the sample was positioned 0.5 mm between the parallel plates and the supporting platform. The storage modulus (G′), loss modulus (G″), and loss factor (tan δ = G″/G′) were obtained.

### 2.9. Thermal Properties (DSC)

Following the method of Hui et al. [12] with modifications, 2 mg of corn flour sample was accurately weighed, and 6 μL of deionized water was added. The sample was sealed in an aluminum pan and kept at 4 °C for 6 h. Afterward, it was removed and placed in the instrument for testing. The testing parameters were set as follows: a heating range of 20 °C to 150 °C and a heating rate of 10 °C/min. Using an empty pan as a reference, the onset temperature (*T_0_*), peak temperature (*T_p_*), conclusion temperature (*T_c_*), and pasting enthalpy change (*∆H*) were obtained.

### 2.10. Pasting Characteristics (RVA)

The pasting properties of the samples were evaluated using a Rapid Visco Analyzer (Rapid-20, Shanghai Baosheng Industrial Development Co., Ltd., Shanghai, China). The testing conditions followed the protocol of Ma et al. [13]. The sample powder was mixed with deionized water to prepare a 10% (*w*/*w*) suspension, which was then placed in the device for testing according to the program. Pasting curves were recorded automatically using the equipment. and peak viscosity (PV), trough viscosity (TV), final viscosity (FV), breakdown (BD), and setback (SB) were calculated.

### 2.11. Dough Preparation and Textural Properties

The sample was mixed with distilled water at a ratio of 1:1.5 (*w*:*v*), placed into a dough mixer, kneaded for 15 min, placed in a container, and left to stand at 40 °C for 30 min to obtain the dough. The test was then performed immediately.

Texture profile analysis (TPA) was performed using a texture analyzer (CT3-4500, Brookfield, MA, USA) equipped with a cylindrical probe (TA44 Cylinder, 4 mm D). The test type was a compression test with a target deformation of 50%, a test speed of 1.5 mm/s, a trigger load of 5 g, no fixture, two replicates for each sample, and a data acquisition rate of 100 pps. Five parameters were obtained from the force-time curve: Stickiness, Springiness, Chewiness, Hardness, and Gumminess.

### 2.12. Noodle Preparation and Cooking Quality

The dough was placed into the machine, and the noodles were squeezed out above the pot of boiling water. After boiling for 1 min, the noodles were removed and placed into cold water to set; the noodles were drained and stored at 4 °C.

The cooking quality test of noodles was carried out according to AACC 66-50 and slightly modified according to the method of Wang and Sun et al. [14,15]. Ten noodles of uniform size and thickness were selected, weighed, and recorded as M_1_, then placed into boiling water for 10 min, removed, and placed into cold water. The number of noodles at this time is recorded as N. Filter was used paper to gently absorb the surface moisture. Then, the noodles were dried with hot air at 100 °C for 8 h and weighed and recorded as M_2_. The breakage rate and cooking loss rate are calculated according to Formulas (5) and (6), respectively.

(5) $$\mathrm{Break}\mathrm{age}\;\mathrm{rate}\;\left(\%\right)=\frac{\left(N-10\right)}{10}\;\times \;100\%$$

(6) $$\mathrm{Cooking}\;\mathrm{loss}\;\mathrm{rate}\;\left(\%\right)=\frac{\left(M_{1}-M_{2}\right)}{M_{1}}\;\times \;100\%$$

### 2.13. In Vitro Digestibility of Noodles

The in vitro digestion characteristics of the noodles were measured with modifications to the protocols by Englyst and Liu [16,17]. Briefly, 0.6 g of chopped cooked noodles were placed in a centrifuge tube containing 5 mL of HCl-KCl buffer solution (0.01 M, pH = 1.5, with 10 mg/mL pepsin). The mixture was incubated at 37 °C with shaking for 30 min (200 rpm). Next, 5 mL of NaOH solution (0.01 M) was added to neutralize the reaction, followed by the addition of 15 mL of sodium acetate buffer (0.01 M, pH = 6.8), which was gently mixed to stop the enzyme reaction. The mixed enzyme solution (5 mL, containing 250 U/mL porcine pancreatic α-amylase and 3000 U/mL glucoamylase) was added and mixed, followed by 1 mL of trypsin solution (1 mg/mL). The mixture was then shaken at 37 °C for an additional 3 h (200 rpm). At time points of 0, 20, 60, 120, 150, and 180 min, 100 μL of the reaction mixture was removed and added to a centrifuge tube containing 1 mL of anhydrous ethanol to stop the enzyme reaction and then centrifuged at 8000 rpm for 15 min.

The glucose content in the digest at each time point was measured using a Glucose content kit (GOPOD oxidase method, BL863A/B, Beijing Lanjieke Technology Co., Ltd., Beijing, China). following the provided instructions and formulas, and the in vitro digestion rate curve was plotted. Each sample was measured in triplicate to eliminate errors.

The content of rapidly digestible starch (RDS), slowly digestible starch (SDS), and resistant starch (RS) in the samples was calculated based on G_0_ (glucose content at 0 min), G_20_ (glucose content at 20 min), and G_120_ (glucose content at 120 min) using Formulas (7)–(9), respectively.

(7) $$\mathrm{RDS}\;\left(\%\right)=\left(G_{20}-G_{0}\right)\;\times \;0.9\;\times \;100\%$$

(8) $$\mathrm{SDS}\;\left(\%\right)=\left(G_{120}-G_{20}\right)\;\times \;0.9\;\times \;100\%$$

(9) $$\mathrm{RS}\;\left(\%\right)=100\%-\mathrm{RDS}-\mathrm{RS}$$

### 2.14. Volatile Flavor Compounds in Noodles

The volatile flavor compounds in the noodles were determined using HS-SPME/GC-MS with modifications based on the method by Wang et al. [18].

The cooked noodles were frozen at -80 °C for 4 h, then cut into small pieces and quickly placed in a liquid nitrogen container for 30 min before being ground into a powder. A sample of 2 g of the powder was mixed with 0.03 mL of ortho-dichlorobenzene (50 mg/L, internal standard) in a 15 mL headspace vial and extracted with a 75 μm CAR/PDMS extraction fiber at 50 °C for 30 min. Subsequently, GC-MS (TQ8050NX, Shimadzu Global Laboratory Consumables Co., Ltd., Kyoto, Japan). was used for thermal desorption at 250 °C for 5 min. Separation was performed using a DB-5MS capillary column (60 × 0.32 mm, 1 μm) with helium as the carrier gas at a flow rate of 0.8 mL/min. The custom conditions were as follows: the initial column temperature was set to 40 °C, held for 2 min, increased to 180 °C at a rate of 5 °C/min, followed by an increase to 250 °C at a rate of 10 °C/min and maintained for 10 min. The mass spectrometry conditions included an ion source temperature of 200 °C, electron energy of 70 eV, and a current of 150 μA.

The volatile compounds were qualitatively identified by comparing their retention indices (RI) and matching the recorded mass spectra stored in the GC-MS data system library.

### 2.15. Statistical Analysis

Unless otherwise specified, each sample was measured at least three times to eliminate errors, and the results are expressed as mean ± standard deviation (SD). A one-way analysis of variance (ANOVA) with Duncan’s test was performed using SPSS 24 (IBM Corporation, Armonk, NY, USA.) to check for significant differences between samples (*p* < 0.05). Graphs were generated using Origin Pro 2024 (OriginLab Corporation, Northampton, MA, USA). and GraphPad Prism 9 (GraphPad Software, Boston, MA, USA).

## 3. Result and Discussion

### 3.1. Amylose/Amylopectin Content

The changes in amylose/amylopectin content are shown in Table 1. It can be observed that different modification methods significantly affect the starch content. After fermentation modification, the proportion of amylose decreases while the proportion of amylopectin increases. This may be due to the degradation of amylose from the amorphous regions under the action of enzymes and organic acids secreted by Lactobacillus plantarum, which are converted into soluble molecules, leading to corresponding changes in the contents of amylose and amylopectin [19]. After combined modification with HMT, the amylose content continued to decrease, and the amylopectin content decreased to a level similar to that of the UM group. This may be because HMT further disrupts both amylose and amylopectin on top of fermentation, leading to a decrease in their content [20]. The results indicate that both fermentation modification and its combination with HMT can significantly affect the changes in starch content.

### 3.2. Color Characteristics

The color characteristics results are shown in Table 1. L* represents the brightness of the sample (with higher values indicating a closer approach to white), a* represents the color change along the red-green axis (with negative values indicating green and positive values indicating red), and b* reflects the color change along the yellow-blue axis (with higher values indicating more yellow). As seen from Table 1, the L* value of the FM group is close to that of the UM group after fermentation modification, while the L* value of the CM group decreases. Both the a* and b* values of all samples show the same trend. Although the a* value was negative (indicating a greenish color), the absolute values of a* and b* decreased significantly in the FM group after fermentation modification, resulting in lighter green and yellow colors. In the CM group, the absolute values of a* and b* significantly increase, nearly returning to values similar to those before modification. The color change in the FM group may be related to the impact of the low pH environment on pigments during fermentation [21]. In the CM group, this change could be attributed to the Maillard reaction under high-temperature conditions, which produces dark-colored substances, leading to a decrease in the L* value and an increase in the absolute values of a* and b*. Similar changes have also been observed in HMT-modified taro starch [22].

### 3.3. Particle Size Distribution

**Figure 2:**
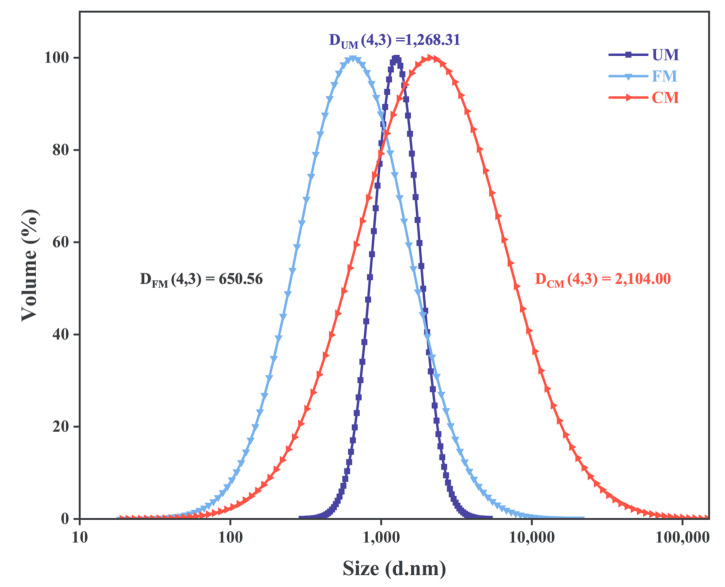
Particle size distribution of the UM, FM, and CM.

The particle size distribution is presented in Table 1 and Figure 2. All samples exhibited a single-peak normal distribution. The FM group’s curve shifted toward smaller particle sizes, with an increased peak width, while the CM group’s curve, based on the FM group, shifted toward larger particle sizes, also showing an increase in peak width. The D(4,3) and D(0.5) values for the three samples were close, ordered as CM > UM > FM, indicating that fermentation modification not only reduced the starch particle size but also increased particle variability. This may result from enzymatic degradation of starch particles by microorganisms during fermentation [23]. In contrast, HMT disrupted the starch particle structure, increasing particle fragments; the starch particles melted and reaggregated, resulting in a larger particle size and broader curve range [24]. Previous studies by Ma et al. [25] and Hu et al. [26] have shown that incorporating smaller starch particles or reducing the size of raw material particles can enhance the processing performance of dough and noodles. This improvement may be attributed to the small starch particles’ tendency to hydrate readily during processing and their rapid gelatinization at high temperatures, which increases system viscosity, making noodles softer and more elastic during cooking. This explains the enhanced cooking tolerance of noodles after HMT treatment.

It should be noted that the dispersion state of the sample in the liquid medium can affect the capture of light scattering signals, and therefore, it is necessary to further analyze the sample in conjunction with microscopic images.

### 3.4. Micromorphology

**Figure 3:**
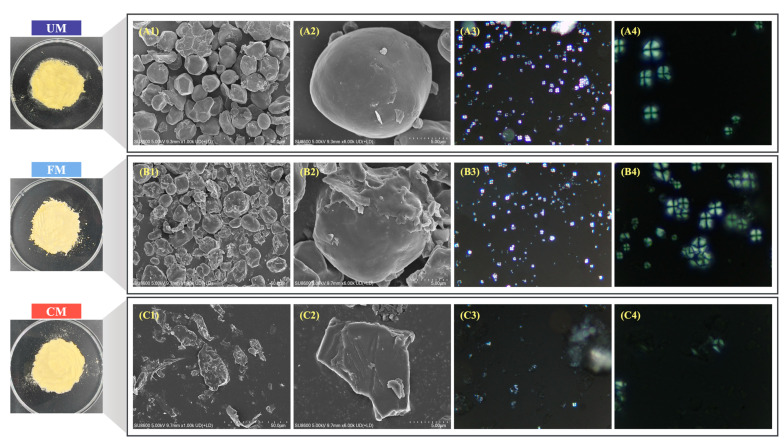
Starch physical appearance, SEM (1.0 k/6.0 k), Polarizing microscope images of UM (**A1**–**A4**), FM (**B1**–**B4**), and CM (**C1**–**C4**).

Figure 3 presents the physical appearance of corn flour and SEM and Polarizing microscope images of starch granules. In the SEM images (A1/A2), the UM group shows starch granules with smooth, crack-free surfaces and complete polyhedral shapes (some spherical), with uniform particle size, clear edges, and minimal fragmentation. The presence of a typical Maltese cross pattern (A3/A4) indicates a high level of internal structural order [27].

Following fermentation modification (B1/B2), the FM group starch granules exhibit roughened surfaces with pits, irregular edge breakage, and agglomeration, suggesting that enzymes and organic acids produced by microorganisms during fermentation disrupted the granule surface structure [28]. The appearance of numerous smaller Maltese crosses (B3/B4) implies that the internally ordered structure of the starch granules was only mildly affected by the fermentation modification. The increase in these smaller Maltese crosses may result from the production of more short-chain starch branches during fermentation [29], consistent with the previously observed increase in amylopectin content.

After HMT treatment (C1/C2), the starch granules in the CM group lost their spherical structure and were replaced by larger irregular shapes. Combined with the aforementioned increase in particle size, we speculate that the starch granules gelatinized, swelled, and melt-polymerized to form irregular aggregates under high temperatures and high moisture content, which is consistent with the research results of Zhong et al. [30]. The disappearance of the Maltese cross pattern (C3/C4) indicates that HMT significantly disrupted the crystalline structure of the starch, reducing its internal order. This irreversible damage is closely associated with enhanced starch functionalities, such as gelatinization, gelation, and solubility [31].

### 3.5. Solubility and WHC

**Figure 4:**
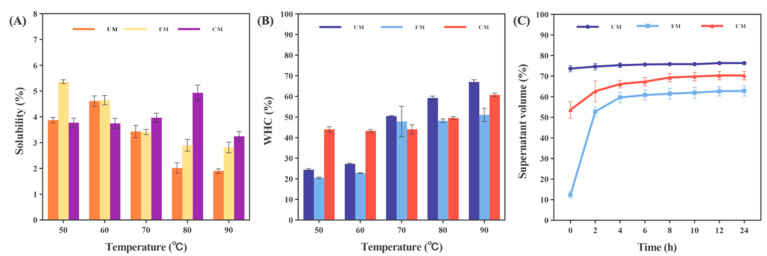
Solubility (**A**) and WHC (**B**) at different temperatures (50–90 °C) and the Retrogradation properties (**C**) at different time points (0–24 h) of UM, FM, and CM.

Figure 4 A and B shows the solubility and water holding capacity (WHC) of the samples at 50–90 °C, respectively. As shown in the figure, the solubility of the UM group increases first and then decreases with increasing temperature, and the solubility is the highest at 60 °C. WHC increases continuously with increasing temperature, which may be because high temperature promotes the efficiency of water molecules binding to polar groups on starch chains, resulting in an increase in WHC, but starch granules also absorb water and swell, limiting dissolution, resulting in a decrease in solubility [32].

After fermentation modification, the solubility of the FM group decreases continuously with increasing temperature, while WHC increases continuously with increasing temperature. However, within the test temperature range, the solubility is greater than that of the UM group, the WHC is less than that of the UM group, and the growth rate slows down after 80 °C. This may be due to the destructive effect of fermentation on the structure of starch granules, which leads to a decrease in the number of polar group sites binding to water molecules and an increase in the amount of starch released, thereby increasing the solubility and reducing the WHC. As the temperature rises, cross-linking rearrangement occurs inside the starch granules, WHC tends to be stable, and starch leaching is limited [28].

After combined modification with HMT, the solubility and WHC of starch do not change significantly in the range of 50–70 °C, showing an enhanced tolerance to this temperature range. When the temperature approaches the HMT treatment temperature, the solubility first increases and then decreases with the increase in temperature, reaching a maximum of 80 °C. WHC also shows a gradual increase trend in the higher temperature range. This may be because HMT causes starch granules to melt and reorganize, improves heat resistance, and reduces the number of polar group sites available for water molecules to bind. Only when the temperature is close to the HMT temperature can the solubility and WHC be affected [33]. When the temperature exceeds the tolerance range, the starch granules will melt and reorganize again, the degree of intermolecular cross-linking increases, and the leaching of starch is limited, resulting in a decrease in solubility [34].

In general, the above results show that fermentation modification can help improve the starch dissolution of corn flour at room temperature or in a slightly hot environment, help improve dough viscosity and facilitate dough forming, and combined modification with HMT can significantly improve the heat resistance of corn flour on this basis, thereby obtaining pasta products with better cooking quality.

### 3.6. Retrogradation Properties

Retrogradation of starch refers to the process in which starch spontaneously transforms from a disordered state (thermodynamically unstable high-energy state) to an ordered state (thermodynamically stable low-energy state) after gelatinization. This process typically occurs during the storage of starchy foods and significantly affects their textural properties, stability, and digestibility [35].

The gel obtained after starch gelatinization will undergo syneresis when left at room temperature or low temperature. This process is related to the structural differences in starch molecules (the degree of polymerization of amylose, the length of amylopectin chains, and the proportion of short chains) [36]. Therefore, the approximate degree of starch retrogradation can be reflected by measuring the change in the proportion of the exuded liquid. The stronger the retrogradation ability, the greater the degree of syneresis, and the more water that is exuded [37,38].

As shown in Figure 4C, the order of the retrogradation properties of the samples throughout the entire period is UM > CM > FM. The retrogradation degree of the UM group increased rapidly within the first 0 to 2 h and remained higher than that of the FM and CM groups throughout the observation period. This may be due to the higher amylose content in the UM group, which makes it more prone to recrystallization and retrogradation after gelatinization [37]. After fermentation modification, the retrogradation degree of the sample significantly decreased. This could be because fermentation disrupted the starch granule structure and reduced the amylose content, weakening its recrystallization ability. After combined modification with HMT, although the degree of retrogradation in the CM group increased, it still exhibited stronger anti-retrogradation ability compared to the UM group. Based on the previously discussed solubility and WHC results, it is evident that combined modification with HMT can counteract the overall decrease in WHC caused by fermentation and improve the anti-retrogradation ability of corn flour.

### 3.7. Long-Range Ordered Structure of Starch

The crystal structure and crystallographic information reflected in the XRD spectra illustrate the impact of different modification methods on the long-range ordered structure of starch.

**Figure 5:**
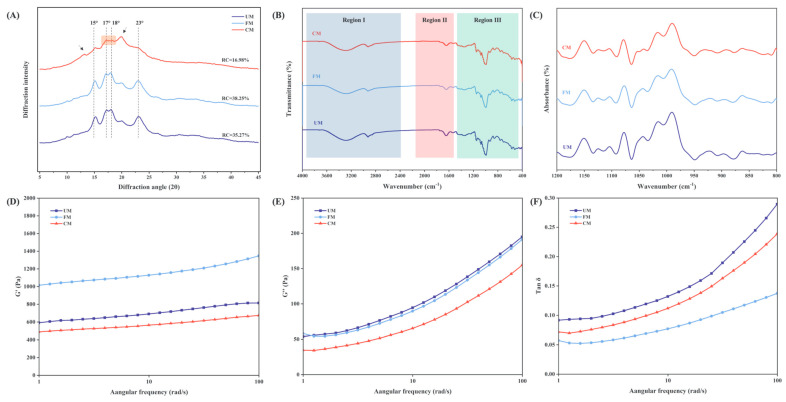
XRD (**A**), FTIR in the range of 4000–400 cm^-1^ (**B**), FTIR deconvolution curves in the range of 1200–800 cm^-1^ (**C**), Storage modulus G′ (**D**), Loss modulus G″ (**E**) and Loss factor Tan δ (**F**) of UM, FM, and CM.

As shown in Figure 5A and Table 2, all samples exhibit prominent characteristic diffraction peaks at 2θ angles of 15°, 17°, 18°, and 23°, displaying the typical A-type crystalline structure of cereal starches [39]. This suggests that neither fermentation modification nor its combination with HMT altered the starch crystal type. However, the data reveal a trend of decreased peak intensity in the CM group. Specifically, the diffraction peak at 17°–18° (highlighted by the shaded area) shows a significant reduction in intensity in the CM group, while the peaks at 12.94° and 19.93° show marked increases (as indicated by arrows). This may be due to HMT disrupting the crystalline structure of starch molecules, promoting a transformation from the A-type to A+V-type crystalline structure, which could be associated with the enhanced thermal stability of the CM group [40].

The relative crystallinity (RC) of starch granules is primarily related to the orderly arrangement of double helices formed by amylopectin side chains [41]. The RC of the samples follows the order FM > UM > CM. The RC of the UM group (35.27%) is close to the RC of native waxy corn starch reported in other studies [42]. After fermentation modification, the RC of the FM group increased to 38.25%, consistent with findings by Zhao et al. [43], who reported that fermentation preferentially utilizes the less dense amorphous regions of starch granules, resulting in increased RC. This is also in line with Zhang et al. [23], who observed that fermentation degrades long-chain starch into more short-chain starch, leading to a short-term dynamic increase in RC. Following combined modification with HMT, the RC of the CM group decreased to 16.98%, possibly due to high temperatures causing degradation of both amylose and amylopectin, leading to a reduction in their content and, thus, a decrease in RC. A similar phenomenon has been observed in DHT (Dry Heat Treatment) processes and is associated with temperature variations [12].

### 3.8. Short-Range Ordered Structure of Starch

FTIR analysis of changes in the short-range order of starch molecules can be divided into three regions: Region I (4000–2500 cm^−1^), corresponding to X-H stretching vibrations (where X = O, N, C, S); Region II (2000–1500 cm^−1^), corresponding to double-bond stretching vibrations; Region III (1500–400 cm^−1^), the fingerprint region, involving C-O, C-C, and C-O-H stretching and bending vibrations.

As shown in Figure 5B, characteristic carbohydrate peaks are observed for all samples within the range of 4000–400 cm^−1^, with consistent peak shapes and no new peaks appearing or disappearing, indicating that the functional groups of corn flour remain largely unchanged after fermentation modification and combined modification with HMT. In Region I, all samples exhibit a broad and strong absorption peak between 3600–3000 cm^−1^, corresponding to the O-H stretching vibration. The absorption peak positions of the UM and FM groups are similar in this region (both at 3278 cm^−1^), while the CM group shifts to 3290 cm^−1^ with decreased peak intensity, possibly due to HMT reducing hydrogen bonding forces and increasing disorder [44]. No significant changes occur in the C-H antisymmetric stretching vibration at 2925 cm^−1^, suggesting limited effects of fermentation and HMT on hydrocarbons. In Region II, no noticeable peak shifts are observed; however, in Region III, the CM group’s absorption peak shifts from 1336 cm^−1^ to 1364 cm^−1^, suggesting that HMT may enhance C-O or C-N stretching vibrations.

Further deconvolution of the FTIR spectra within the range of 1200–800 cm^−1^ was conducted, analyzing absorbance ratios at 1047 cm^−1^ (C-O-C stretching, sensitive to crystalline regions), 1022 cm^−1^ (C-O-H stretching, sensitive to amorphous regions), and 995 cm^−1^ (associated with α-1,4 and α-1,6 glycosidic bonds, sensitive to hydrogen bond structure) [45]. These ratios, designated as R (1047/1022) and R (995/1022), were used to represent changes in short-range order and double-helical content, respectively [46]. As shown in Figure 5C and Table 2, the deconvoluted spectra display consistent peak shapes across all samples. The R (1047/1022) ratio, reflecting short-range order in starch molecules, continuously decreases after modification (UM > FM > CM), indicating a reduction in short-range order. This may be attributed to fermentation affecting the crystalline-to-amorphous ratio and the combined modification with HMT, inducing molecular chain breakage and structural disruption. The absorption peak around 995 cm^−1^, representing the amorphous region, shows a peak intensity and R (995/1022) order of FM > UM > CM, consistent with the previously discussed amylopectin content and RC order, reflecting changes in double-helical content. The increase in R (995/1022) for FM after fermentation may be related to the degradation of long-chain amylopectin side chains into more short chains, facilitating the reformation of double helices [47]. Organic acids and microbial metabolites produced during fermentation may also influence hydrogen bonding between starch molecules, thus stabilizing the double-helical structure. After combined modification with HMT, the decrease in R (995/1022) indicates that moisture and heat disrupt double-helical structures, leading to starch molecular degradation or reorganization.

### 3.9. Dynamic Rheological Properties

Dynamic rheology is used to describe the interaction between polymers in the dough network and between polymers and reinforcing fillers, making it an important parameter for evaluating product quality [48]. Figure 5D–F shows the relationship between G′ (storage modulus), G″ (loss modulus), and Tan δ (loss factor) of the sample and the angular frequency, respectively. G′ and G″ reflect the changes in elasticity and viscosity of the sample within the scanning frequency range. When G′ > G″, it behaves as a viscoelastic solid, and when G″ > G′, it behaves as a viscoelastic fluid. Tan δ is the ratio of G″ to G′, indicating the dynamic change in the ratio of elasticity to viscosity in the system. When 0 < Tan δ < 1, the system is a viscoelastic solid, and Tan δ > 1 indicates a viscoelastic fluid. The smaller the Tan δ, the stronger the elasticity and the worse the fluidity [49].

As shown in the figure, G′, G″, and Tan δ of all samples increase with the increase in frequency, showing a correlation with frequency. Additionally, G′ (FM > UM > CM), G″ (UM > FM > CM), and Tan δ (UM > CM > FM) follow a specific ranking order. In the entire scanning frequency range, G′ > G″ and 0 < Tan δ < 1, indicating that all samples exhibit a tendency to behave as viscoelastic solids. These relatively solid gel systems exhibit recoverable deformation and display typical weak gel properties [50].

After fermentation modification, the G′ of the FM group is significantly higher than that of the UM group, but G″ does not differ much between the two, and Tan δ decreases. This indicates that fermentation improves elasticity more than viscosity and effectively increases the degree of internal cross-linking. This improvement may be due to fermentation causing long-chain starch to depolymerize into a large amount of short-chain starch. After gelatinization and water absorption, the short-chain starch undergoes molecular rearrangement and shows a recrystallization effect, enhancing the internal cross-linking degree and structural strength of the starch gel network. As a result, the elasticity and structural stability of the starch gel is increased [51].

After combined modification with HMT, both G′ and G″ were significantly reduced and were lower than in the UM group, while Tan δ was greater than in the FM group but still lower than in the UM group. This may be because high temperature destroyed the structure of starch granules, and water infiltration reduced the structural stability, weakening the strength of the gel network [52]. However, at this point, the degree of internal cross-linking was still greater than in the UM group, contributing to improved processing stability of the product.

### 3.10. Thermal Properties

**Figure 6:**
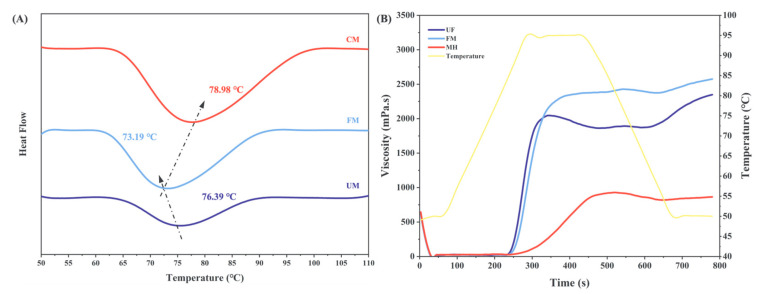
DSC curves (**A**) and RVA curves (**B**) of the UM, FM, and CM.

The DSC data for the samples are presented in Table 3 and Figure 6A. After fermentation modification, the *T_0_* (onset temperature) and *T_P_* (peak temperature) of the FM group decreased, while *T_C_* (conclusion temperature) was similar to that of the UM group. Additionally, Δ*T* (*T_C_ − T_0_*) and Δ*H* (enthalpy of gelatinization) increased. These changes can be attributed to the depolymerization of long-chain amylopectin during fermentation and the increase and rearrangement of short-chain amylopectin, which form a more complex short amylopectin structure [53]. The higher amylopectin content results in a lower gelatinization temperature. The increase in double helicity caused by higher amylopectin content requires more heat for gelatinization [54]. Consequently, the FM group shows a decrease in *T_P_* and an increase in Δ*T* and Δ*H*.

After combined modification with HMT, the *T*_0_ of the CM group was improved compared with the FM group and approached the value of the UM group. In addition, *T_P_*, *T_C_*, and Δ*H* increased, while Δ*T* was similar to that of the FM group. This indicates that HMT helped repair the lower gelatinization temperature caused by fermentation, requiring more heat for gelatinization and enhancing thermal stability [55]. These findings are consistent with the results observed in the solubility and WHC analyses. Overall, the CM group exhibited stronger heat-resistant processing potential.

### 3.11. Gelatinization Behavior

Gelatinization is a thermal process where starch granules absorb water and expand upon heating in an aqueous solution. This process is typically divided into three stages [56]:

1. Reversible water absorption stage: In this stage, slight expansion occurs at the beginning of heating. A suspension forms when stirred, but precipitation occurs when left to stand, with no change in starch birefringence;
2. Irreversible water absorption stage: As temperature increases, chemical bonds within the starch molecules break, the crystalline regions loosen, and the rate of water absorption and expansion increases rapidly. During this stage, starch birefringence disappears;
3. Complete disintegration stage: In this final stage, starch granules melt and disintegrate, releasing internal starch molecules that interconnect and entangle to form a gel network.

The RVA results for the UM, FM, and CM groups are presented in Table 3 and Figure 6B, where parameters such as Peak Viscosity (PV), Trough Viscosity (TV), Final Viscosity (FV), Breakdown (BD), and Setback (SB) are analyzed. The results indicate that compared to the UM group, the PV, TV, and FV of the FM group increased while the BD and SB decreased. This enhancement in viscosity during starch gelatinization can be attributed to the increased proportion of amylopectin in the system, which subsequently increased viscosity and limited breakdown, while the increase in amylose content may have the opposite effect [57]. The fermentation process altered the amylose/amylopectin ratio, which may account for the improved gelatinization properties of the FM group. In contrast, the CM group showed a decrease in PV, TV, FV, BD, and SB compared to the FM group, indicating that the HMT treatment weakened the viscosity of the system. This result is consistent with the findings for the loss modulus (G″) mentioned earlier. High temperatures trigger the degradation of starch granules, causing them to melt and disintegrate. The resulting reorganization leads to the formation of an irregular structure, similar to what is shown in Figure 3 [58]. This reduction in viscosity post-HMT treatment helps the product form a more solid texture, improves its heat resistance, and enhances its anti-retrogradation properties [59].

### 3.12. Textural Properties of Dough

As shown in Table 4, compared to the UM group, the FM and CM groups demonstrated significantly improved texture properties, including stickiness, springiness, chewiness, hardness, and gumminess, with the CM group exhibiting the best results (CM > FM > UM). The improvements in texture properties in the FM group are attributed to the disruption of the long-branch starch structure and the formation of a more complex short-branch starch structure through fermentation. The combined modification with HMT further enhanced these properties, likely due to the melting and rearrangement of starch molecules, which resulted in the formation of a tighter network structure [60].

Natural corn flour, lacking gluten protein, struggles to form a stable and solid dough, resulting in a loose structure with insufficient stickiness, making it unsuitable for producing high-quality noodles [61]. Improving the texture properties of dough is essential for enhancing the processing performance and quality of noodles. Key texture attributes, such as stickiness, springiness, chewiness, and hardness, directly affect the quality of noodles. For instance, increased stickiness helps prevent noodles from breaking or deforming during cooking, while good springiness ensures a smooth texture and reduces the likelihood of clumping during prolonged heating. Increased chewiness results in a firmer texture, and high hardness improves the ductility and compressive strength of dough [62]. In conclusion, the dough from the CM group displayed superior texture properties and processing stability, making it more suitable for producing higher-quality noodles.

### 3.13. Cooking Properties of Noodles

The cooking quality of noodles is an important parameter, typically assessed by the breakage rate (the proportion of noodles that break during cooking) and the cooking loss rate (the amount of dry matter lost during cooking) [63]. As shown in Table 4, the rankings of the breaking rate and cooking loss rate are CM < FM < UM, indicating that both fermentation modification and combined fermentation modification with HMT improved the structural integrity and cooking quality of the noodles. This improvement can be attributed to the fact that both fermentation and HMT contribute to the formation of a stronger gel network, thereby limiting the leaching of soluble components. These findings are consistent with the results reported by Mo and Chandla [64,65]. Additionally, the organic acids produced during fermentation may further contribute to the thickening of the system [66]. Overall, the enhancement of cooking quality aligns with the TPA results, suggesting that fermentation modification improves the processing quality of corn dough, while the combined fermentation and HMT modification play a critical role in determining the quality of TFCN.

### 3.14. In Vitro Digestive Properties of Noodles

The starch digestion process involves the breakdown of glycosidic bonds in the starch chain by digestive enzymes, ultimately releasing glucose. Based on the rate of glucose release, starch components can be categorized into rapidly digestible starch (RDS), slowly digestible starch (SDS), and resistant starch (RS) [67]. Foods rich in SDS and RS are considered low glycemic index (GI) foods, which can help prevent metabolic diseases like diabetes and have drawn considerable research interest [68]. On the other hand, foods with high RDS content can quickly replenish energy, making them suitable for hypoglycemic patients [69].

**Figure 7:**
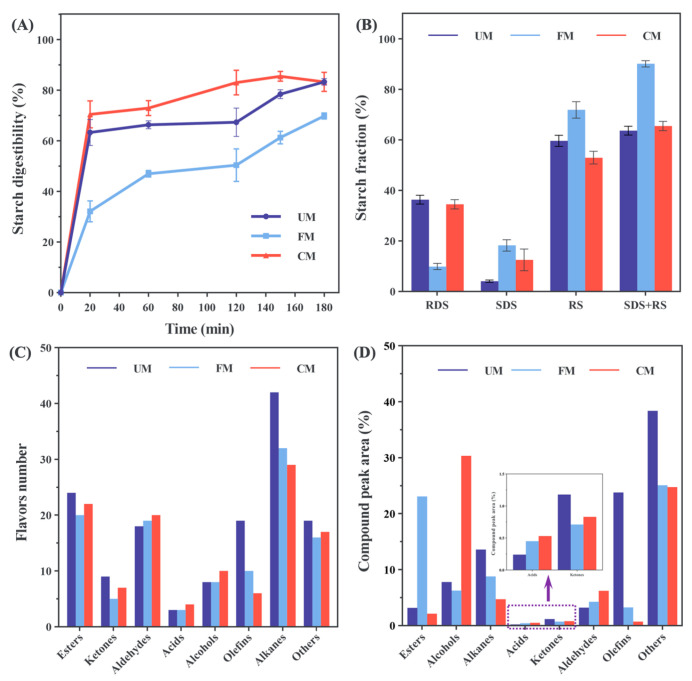
In vitro digestibility (**A**), nutrient composition (**B**), flavoring substance species (**C**), and relative peak area share (**D**) for UM, FM, and CM.

As shown in Figure 7 A and B, all samples exhibited a rapid increase in glucose release during the first 20 min of digestion, followed by a slower increase after 20 min. The digestion resistance, RDS, SDS, RS, and SDS + RS contents followed the order: FM > UM > CM for digestion resistance, and UM > CM > FM for RDS, with FM showing the highest SDS and RS content, and CM having the lowest. Notably, noodles from the FM group showed lower digestibility across the entire digestion stage compared to the UM group. This could be because, during fermentation modification, microorganisms preferentially utilize free sugars in the fermentation broth, reducing the detectable glucose content. Additionally, the rearrangement of short amylopectin into a crystalline structure lowers the substances that promote rapid blood sugar increase, leading to reduced RDS content and improved digestibility in the later stages, as evidenced by a higher SDS + RS ratio. Consequently, the FM noodles exhibited characteristics typical of low-GI foods.

In contrast, the noodles from the CM group exhibited higher digestibility compared to both the UM and FM groups throughout the digestion process. This may be due to the high temperature and moisture content of the combined modification, which disrupts the starch granules, exposing more sites for enzymatic hydrolysis, making the starch more susceptible to digestion, and resulting in higher RDS content. This finding is consistent with the research by Chen et al. [39]. The CM noodles also showed a higher SDS + RS content than the UM group, which can be attributed to the higher SDS content in the CM noodles, helping to balance the digestion characteristics.

These results highlight that modification does not always result in low GI properties for starch, as factors such as enzyme accessibility and starch structure influence this outcome [70]. Nonetheless, it is possible to create foods with both high and low GI properties by adjusting the modification method and conditions to control the interaction between starch and digestive enzymes [71].

### 3.15. Flavor Substances in Noodles

The types and relative peak area results of volatile flavor compounds in noodles are illustrated in Figure 7 C and D, with specific compound names and contents listed in Supplementary Materials. A total of 224 volatile compounds were detected through HS-SPME-GC-MS, including esters (45), ketones (9), aldehydes (25), acids (7), alcohols (19), olefins (23), alkanes (65), and others (31). Among these, the UM group noodles contained 142 types, the FM group noodles contained 113 types, and the CM group noodles contained 115 types. Notably, the FM group noodles generated 42 new aroma compounds, and the CM group noodles generated 40 new aroma compounds.

Esters are responsible for fruity (short-chain esters) and fatty aromas (long-chain esters) [72]. All three types of noodles contained methyl enanthate (fruity aroma), isopropyl palmitate (creamy aroma), and 2-ethylhexyl salicylate (sweet citrus and floral aroma). After fermentation modification, the content of 2-phenylethyl acetate (floral aroma) significantly increased to 1.140 μg/kg. The fermentation process also led to the formation of homosalate, contributing a mild mint aroma to the noodles in the FM group, while in the CM group, the unique nerol acetate generated a refreshing citrus and sweet pea aroma.

Ketones contribute grassy, creamy, and floral aromas [73]. 2(3H)-Furanone, di-hydro-5-pentyl (coconut aroma), was detected in all noodle samples. Although 7,9-di-tert-butyl-1-oxaspiro (4,5) deca-6,9-diene-2,8-dione (herbal and spicy aroma) was present in all three noodle groups, its content significantly decreased to 0.081 μg/kg in the CM group, indicating poor thermal stability. Additionally, 2-undecanone (fruity aroma) and thujone (grassy aroma) were only detected in UM group noodles and can be used as characteristic compounds to distinguish the FM and CM group noodles.

Aldehydes, including hexanal, nonanal, heptanal, and valeraldehyde, are key compounds that contribute to the flavor of fermented cereal products [74]. These aldehydes may be produced by the hydrolysis of enzymes secreted by lactic acid bacteria or the oxidation of free fatty acids. Our results also detected these aldehydes, consistent with previous studies [18], and they form the aromatic base in noodles, contributing vegetable, fatty, citrus, and fruit aromas. The contents of nonanal (3.895 μg/kg, pea aroma) and 2,4-decadienal (E,E) (1.291 μg/kg, fruit aroma) in the UM group noodles were significantly higher than those in the FM and CM groups. This suggests that fermentation and HMT contribute to the degradation of these aldehydes. 2-Furancarbaldehyde (5-methyl) (spicy flavor), produced during fermentation, was also degraded after HMT. 2,4-Decadienal (E,E) (fruit aroma) and 2-nonenal (E) (citrus aroma) were highest in the CM group, indicating that they may have better thermal stability.

Regarding acids and alcohols, fermentation and its combined modification with HMT promoted the increase in caprylic acid (fruity aroma). Nonanoic acid (cheese flavor), generated after fermentation, disappeared after HMT. Three new acid compounds found in the CM group noodles—tetradecanoic acid, 9,12-octadecadienoic acid (Z,Z), and caproic acid—added fatty and subtle plant aromas. Alcohols in noodles can arise from carbohydrate metabolism, amino acid dehydrogenation, and decarboxylation reactions, typically contributing fruity, nutty, floral, and sweet aromas [75]. Phenylethanol and linalool, which have rose and citrus aromas, showed opposite trends before and after fermentation. Phenylethanol increased from 2.158 μg/kg to 9.412 μg/kg, while linalool decreased from 20.50 μg/kg to 6.853 μg/kg. Both were common to all three noodle types. Additionally, the newly generated levomenthol, after fermentation, added a refreshing minty flavor to the FM group noodles. After fermentation, (E)-β-farnesene (woody aroma) and 1,3,6-octatriene, 3,7-dimethyl-, (Z) (floral and herbal aroma) disappeared. β-Myrcene (spicy flavor) and humulene (woody aroma) disappeared after HMT. The newly generated caryophyllene oxide in the FM group noodles contributed a refreshing spicy or woody aroma, enriching the overall aroma of the noodles. Notably, D-limonene (lemon aroma), which is unique to the UM group noodles and present at a high content (62.397 μg/kg), disappeared after fermentation modification, aligning with the findings of Mockus et al. [76] and suggesting its association with the fermentation process.

The UM group noodles contain a relatively high amount of long-chain alkanes, contributing a heavy, greasy, and waxy aroma that adds limited value to the overall flavor profile [77]. After fermentation modification and combined modification with HMT, the content of long-chain alkanes generally decreases. For example, hexadecane, a medium-chain alkane with a mild hydrocarbon aroma, is present in all three noodle types but gradually decreases post-modification, with a marked reduction in the CM group noodles. This suggests that fermentation and HMT significantly impact long-chain alkanes.

Additionally, some monoterpenes, unsaturated aldehydes, halogenated compounds, furans, and phenolic compounds were detected. Naphthalene, with a spicy aroma, shows little change in content after fermentation but disappears after HMT, potentially converting to 1,2-dihydro-1,1,6-trimethyl-naphthalene under high temperatures, imparting a licorice aroma to CM group noodles. Furan, 2-pentyl-, which has a fruity aroma, is unique to CM group noodles and is relatively high in concentration (3.637 μg/kg). This compound is likely linked to lipid oxidation, suggesting that HMT may promote lipid oxidation and generate higher concentrations of furan compounds [78].

Further analysis of the flavor content in Supplementary Materials, where ND stands for “not detected”, revealed that the UM group noodles were rich in flavor components, with a high proportion of alkenes and alkanes, commonly associated with the natural aroma of plant-based foods. However, these compounds lack extensive transformation, contributing only a raw and neutral flavor profile. After fermentation modification, FM group noodles showed a significant increase in ester content, with a relative reduction in other flavor compounds. This indicates that fermentation simplifies the aroma composition and enhances characteristic flavor components. After the combined modification with HMT, the proportion of alcohols increased in CM group noodles, while esters and aldehydes were retained, along with newly generated ketones and acids from fermentation. This enriches the noodles’ flavor profile, potentially improving sensory appeal.

## 4. Conclusions

This study investigated the effects of fermentation modification and combined modification with HMT on the multi-scale structure, physical and chemical properties, and TFCN quality of corn flour. The results indicate that fermentation modification primarily improved the physical and chemical properties of corn flour, significantly increasing the amylopectin ratio, relative crystallinity, and short-range order. Although fermentation caused the appearance of pits and cracks on the surface of the starch granules, the starch granules largely maintained their intact structure, and the crystallinity remained unchanged. Fermentation modification also significantly enhanced the anti-retrogradation properties, anti-digestible component content, and ester content of the corn flour. The combined modification of fermentation and HMT further improved the performance of the corn flour. Starch granules underwent melting and reorganization, and the heat resistance of the corn flour was enhanced. The dough texture characteristics and noodle cooking quality showed significant improvements. In particular, the content of anti-digestible starch in noodles remained higher than in the UM group noodles. The levels of aldehydes, acids, and alcohols were significantly increased, while the content of olefins and alkanes was reduced, contributing to the formation of more desirable noodle aromas. There are still some limitations in this study. For example, the impact of different corn varieties (such as sweet corn and waxy corn) on the modification outcomes has not been explored, and it is not yet known whether fermentation modification and its combination with HMT have a universal effect on different corn varieties. In addition, the process parameters involved in fermentation and HMT, such as temperature and time, could affect the stability of the modification results. Another limitation is the lack of an in-depth investigation into the molecular mechanisms underlying the modification process. Therefore, future research could address these limitations by comparing multiple corn varieties, optimizing modification conditions, and exploring the roles of these factors during the modification process using molecular biology techniques. However, overall, the study reveals that fermentation modification significantly enhanced the multi-scale structure and physical and chemical properties of corn flour, while combined modification with HMT further improved the texture characteristics and cooking quality of noodles, building on the benefits provided by fermentation modification. These findings will contribute to improving the product quality of TFCN.

## Figures and Tables

**Table 1:** Amylose and amylopectin content, color characteristics, and median particle size of UM, FM, and CM.

	UM	FM	CM
Amylose (%)	41.61 ± 0.41 ^a^	38.65 ± 0.84 ^b^	35.18 ± 0.44 ^c^
Amylopectin (%)	47.40 ± 1.18 ^b^	54.62 ± 0.81 ^a^	47.39 ± 0.73 ^b^
L*	91.19 ± 0.27 ^a^	91.33 ± 0.05 ^a^	88.07 ± 0.11 ^b^
a*	−0.57 ± 0.05 ^b^	−0.33 ± 0.03 ^a^	−0.67 ± 0.07 ^c^
b*	29.38 ± 0.29 ^a^	25.42 ± 0.18 ^c^	28.49 ± 0.49 ^b^
D(0.5) (nm)	1378.90 ± 256.01 ^b^	773.63 ± 116.22 ^c^	1887.28 ± 215.91 ^a^

Data are presented as mean ± standard deviation (n = 3), and values in columns with different superscripts are significantly different (*p* < 0.05). Statistical analysis is based on independently repeated experiments.

**Table 2:** XRD diffraction peak information and FTIR spectrum information of UM, FM, and CM.

	UM				FM				CM			
	15°	17°	18°	23°	15°	17°	18°	23°	15°	17°	18°	23°
Peak position (°2θ)	15.12	17.30	18.29	23.23	15.06	17.28	18.24	23.15	15.08	17.23	18.22	23.00
Peak height (cts)	716.96	892.57	593.72	906.23	709.64	892.38	530.10	858.08	123.43	281.31	194.43	357.38
FWHM (°2θ)	0.89	1.41	0.93	1.64	0.85	1.45	1.02	1.76	0.48	0.97	0.72	2.54
Area (cts*°2θ)	687.15	1353.00	671.80	1772.82	649.83	1390.20	660.36	1767.19	64.14	293.57	183.59	1019.47
R (1047/1022)	1.12 ± 0.11 ^a^				1.10 ± 0.09 ^a^				0.72 ± 0.12 ^b^			
R (995/1022)	2.10 ± 0.05 ^a^				2.18 ± 0.04 ^a^				1.80 ± 0.06 ^b^			

R (1047/1022) and R (995/1022) data are expressed as mean ± standard deviation (n = 3), and values with different superscripts indicate significant differences (*p* < 0.05). Statistical analysis is based on independently repeated experiments.

**Table 3:** DSC thermal properties and RVA gelatinization characteristics of UM, FM, and CM.

	UM	FM	CM
*T*_0_ (°C)	64.70 ± 0.70 ^a^	62.77 ± 0.32 ^b^	64.44 ± 0.18 ^a^
*T_P_* (°C)	76.15 ± 0.34 ^b^	73.55 ± 0.51 ^c^	78.98 ± 0.02 ^a^
*T_C_* (°C)	89.04 ± 1.37 ^a^	89.01 ± 1.35 ^a^	90.71 ± 1.02 ^a^
*T_C_-T*_0_ (°C)	24.34 ± 2.07 ^a^	26.24 ± 1.02 ^a^	26.27 ± 0.84 ^a^
Δ*H* (J/g)	1.62 ± 0.01 ^c^	1.88 ± 0.01 ^b^	2.01 ± 0.01 ^a^
PV (cP)	2022.12 ± 57.19 ^b^	2366.36 ± 91.61 ^a^	596.56 ± 165.16 ^c^
TV (cP)	1809.12 ± 76.23 ^b^	2331.80 ± 108.04 ^a^	576.48 ± 169.93 ^c^
FV (cP)	2194.28 ± 256.74 ^b^	2543.04 ± 88.23 ^a^	680.60 ± 160.66 ^c^
BD (cP)	213.00 ± 20.97 ^a^	34.56 ± 17.58 ^b^	20.08 ± 5.21 ^b^
SB (cP)	385.16 ± 181.35 ^a^	211.24 ± 19.87 ^ab^	104.12 ± 9.28 ^b^

Data are presented as mean ± standard deviation (n = 3), and values in columns with different superscripts are significantly different (*p* < 0.05). Statistical analysis is based on independently repeated experiments.

**Table 4:** TPA textural properties of dough and cooking quality of noodles of UM, FM, and CM.

	UM	FM	CM
Stickiness (mJ)	0.82 ± 0.15 ^b^	1.23 ± 0.15 ^b^	10.36 ± 4.47 ^a^
Springiness (mm)	7.88 ± 1.29 ^b^	9.74 ± 1.02 ^b^	12.67 ± 4.79 ^a^
Chewiness (mJ)	1.71 ± 0.35 ^b^	1.86 ± 0.83 ^b^	13.88 ± 8.64 ^a^
Hardness (g)	50.69 ± 4.70 ^c^	92.07 ± 21.28 ^b^	350.84 ± 52.84 ^a^
Gumminess (g)	17.88 ± 2.18 ^b^	23.47 ± 6.80 ^b^	89.81 ± 79.00 ^a^
Breakage rate (%)	47.00 ± 12.52 ^a^	20.00 ± 11.55 ^b^	6.00 ± 6.99 ^c^
Cooking Loss (%)	6.44 ± 0.68 ^a^	3.17 ± 1.14 ^b^	1.60 ± 0.35 ^c^

Data are presented as mean ± standard deviation (n = 3), and values in columns with different superscripts are significantly different (*p* < 0.05). Statistical analysis is based on independently repeated experiments.

## Data Availability

The original contributions presented in the study are included in the article/Supplementary Materials; further inquiries can be directed to the corresponding author.

## Supplementary Materials

The following supporting information can be downloaded at: https://www.mdpi.com/article/10.3390/foods13244043/s1, Table S1: Volatile components, retention index and relative content of UM, FM, and CM noodle.
