# Supplementary material for: Effects of Fermentation Modification and Combined Modification with Heat-Moisture Treatment on the Multiscale Structure, Physical and Chemical Properties of Corn Flour and the Quality of Traditional Fermented Corn Noodles
Source: Foods. 2024 Dec 14;13(24):4043. doi: 10.3390/foods13244043 (PMC11728366; doi:10.3390/foods13244043)
Supplement: Supplementary file 1 [file foods-13-04043-s001.zip › foods-3345683-supplementary.pdf]

Supplementary Tables in the Manuscript

Effects of Fermentation Modification and Combined Modification with Heat-Moisture Treatment on the Multiscale Structure, Physical and Chemical Properties of Corn Flour and the Quality of Traditional Fermented Corn Noodles

Chen Mao[1], Sijia Wu[2], Ling Zhang[1] and Hong Zhuang[1,*]

[1] College of Food Science and Engineering, Jilin University, Changchun, 130062, China.

[2] Shenzhen Institutes of Advanced Technology, Chinese Academy of Sciences, Shenzhen 518055, China.

*** Correspondence: zhuanghong@jlu.edu.cn;**

College of Food Science and Engineering, Jilin University

Changchun, 130062, P.R. China.

Tel.: 86+13944102716

E-mail: zhuanghong@jlu.edu.cn (Hong Zhuang)

Table S1. Volatile components, retention index and relative content of UM, FM, and CM noodle.

Volatile flavor substances	RI	Relatively content (μg/Kg)		
		UM	FM	CM
Esters				
1,2-Benzenedicarboxylic acid, bis(2-methylpropyl) ester	1908	378.00	296.00	121.00
1,2-Propanediol, 3,3'-oxydi-, tetranitrate	2396	ND	62505.00	ND
1,3-Propanediol, 2,2-dimethyl-, diacetate	1166	226.00	ND	ND
1-Hexanol, 5-methyl-2-(1-methylethyl)-, acetate	1189	ND	ND	307.00
2,2,4-Trimethyl-1,3-pentanediol diisobutyrate	1605	912.00	ND	272.00
2,6-Octadien-1-ol, 3,7-dimethyl-, acetate	1352	128.00	ND	ND
2-Ethylhexyl 2-ethylhexanoate	1651	ND	ND	134.00
2-Ethylhexyl acrylate	1208	368.00	ND	ND
2-Ethylhexyl salicylate	1913	ND	618.00	ND
2-Propenoic acid, 6-methylheptyl ester	1208	ND	423.00	211.00
2-Thiopheneacetic acid, 4-tetradecyl ester	2389	ND	347.00	ND
2-Thiopheneacetic acid, 4-tridecyl ester	2289	ND	ND	132.00
Acetic acid, 2-phenylethyl ester	1259	598.00	1140.00	346.00
Benzoic acid, undecyl ester	2054	135.00	ND	ND
Butanoic acid, tridec-2-ynyl ester	1896	ND	756.00	ND
Carbonic acid, decyl dodecyl ester	2551	160.00	ND	ND
Carbonic acid, decyl hexadecyl ester	2948	702.00	ND	ND
Carbonic acid, decyl tetradecyl ester	2749	ND	ND	347.00
Carbonic acid, octadecyl vinyl ester	2342	540.00	ND	ND
Chloroacetic acid, 2-ethylhexyl ester	1344	ND	105.00	ND
Decanoic acid, ethyl ester	1381	182.00	191.00	306.00
Dibutyl phthalate	2037	550.00	785.00	291.00
Dodecanoic acid, ethyl ester	1580	ND	ND	162.00
Ethyl Oleate	2185	94.00	ND	ND
Glutaric acid, 2-ethylhexyl 3,7-dimethyloctyl ester	2450	116.00	ND	ND
Heptanoic acid, methyl ester	984	628.00	263.00	392.00
Hexadecanoic acid, ethyl ester	1978	186.00	119.00	91.00
Hexanoic acid, 1-ethenyl-1,5-dimethyl-4-hexenyl ester	1670	ND	ND	180.00
Homosalate	2037	ND	256.00	ND
Isopropyl myristate	1814	ND	145.00	ND
Isopropyl palmitate	2013	123.00	106.00	99.00
l-(+)-Ascorbic acid 2,6-dihexadecanoate	4756	224.00	ND	ND
Linalyl acetate	1272	978.00	503.00	665.00
Linoleic acid ethyl ester	2193	124.00	ND	ND
Nerolidyl acetate	1754	ND	ND	242.00
Octanoic acid, ethyl ester	1183	ND	241.00	291.00
Oxalic acid, bis(6-ethyloct-3-yl) ester	2286	ND	ND	262.00
Oxalic acid, cyclohexylmethyl tridecyl ester	2606	1460.00	ND	ND
Oxalic acid, monoamide, N-(2-phenylethyl)-, isohexyl ester	2192	320.00	ND	ND
Sulfurous acid, cyclohexylmethyl hexadecyl ester	2994	ND	ND	470.00
Sulfurous acid, decyl hexyl ester	2235	ND	ND	99.00
Sulfurous acid, decyl pentyl ester	2135	ND	603.00	ND
Sulfurous acid, hexyl octyl ester	2036	1572.00	101.00	409.00
Trichloroacetic acid, tetradecyl ester	2166	ND	93.00	ND
Trichloroacetic acid, tridec-2-ynyl ester	2084	136.00	ND	ND
Ketones				
1-Penten-3-one, 1-(2,6,6-trimethyl-1-cyclohexen-1-yl)-	1557	637.00	ND	422.00
2(3H)-Furanone, dihydro-5-pentyl-	1284	88.00	142.00	143.00

Table S1. (continued)

2-Butanone, 4-(2,6,6-trimethyl-1-cyclohexen-1-yl)-	1449	433.00	256.00	257.00
2-Undecanone	1251	248.00	ND	ND
3-Buten-2-one, 4-(2,6,6-trimethyl-1-cyclohexen-1-yl)-	1457	328.00	ND	211.00
5,9,13-Pentadecatrien-2-one, 6,10,14-trimethyl-, (E,E)-	1902	183.00	103.00	147.00
5,9-Undecadien-2-one, 6,10-dimethyl-	1420	1314.00	1207.00	993.00
7,9-Di-tert-butyl-1-oxaspiro(4,5)deca-6,9-diene-2,8-dione	2081	229.00	252.00	81.00
Thujone	1062	401.00	ND	ND
Aldehydes				
1-Cyclohexene-1-carboxaldehyde, 2,6,6-trimethyl-	1204	164.00	172.00	201.00
2,4-Decadienal, (E,E)-	1220	1291.00	927.00	3124.00
2,4-Decadienal, (E,Z)-	1220	980.00	ND	712.00
2,4-Nonadienal, (E,E)-	1120	127.00	200.00	196.00
2,6-Octadienal, 3,7-dimethyl-, (E)-	1174	98.00	ND	ND
2-Furancarboxaldehyde, 5-methyl-	920	ND	694.00	ND
2-Heptenal, (E)-	913	387.00	410.00	306.00
2-Nonenal, (E)-	1112	1202.00	1413.00	1652.00
2-Octenal, (E)-	1013	ND	1061.00	812.00
2-Tridecenal, (E)-	1510	160.00	ND	ND
2-Undecenal	1313	311.00	271.00	413.00
7-Hexadecenal, (Z)-	1808	208.00	180.00	173.00
7-Tetradecenal, (Z)-	1609	ND	ND	128.00
Benzaldehyde	982	164.00	ND	365.00
Benzeneacetaldehyde	1081	83.00	518.00	227.00
Bicyclo[3.1.1]heptane-2-carboxaldehyde, 6,6-dimethyl-	1126	141.00	ND	ND
Citral	1174	ND	126.00	112.00
Dodecanal	1402	391.00	389.00	332.00
Furfural	831	ND	1035.00	861.00
Heptanal	905	ND	259.00	266.00
Hexanal	806	167.00	647.00	ND
Undecanal	1303	185.00	212.00	250.00
Decanal	1204	689.00	739.00	677.00
Nonanal	1104	3895.00	2838.00	2789.00
Octanal	1005	ND	599.00	490.00
Acids				
.alpha.-Guaiene	1490	135.00	150.00	ND
9(E),11(E)-Conjugated linoleic acid	2183	110.00	ND	ND
9,12-Octadecadienoic acid (Z,Z)-	2093	ND	ND	112.00
Hexanoic acid	974	ND	ND	261.00
Nonanoic acid	1272	ND	478.00	ND
Octanoic acid	1173	543.00	727.00	729.00
Tetracosanoic acid	2763	ND	ND	93.00
Alcohols				
1-Decanol, 2-hexyl-	1790	772.00	ND	305.00
1-Decanol, 2-octyl-	1989	ND	803.00	ND
1-Heptatriacotanol	3942	ND	ND	186.00
1-Nonanol	1159	203.00	ND	ND
1-Octanol	1059	ND	115.00	ND
1-Octanol, 2-butyl-	1393	ND	93.00	97.00
1-Tridecanethiol	1617	379.00	ND	ND
2,5-Dimethylcyclohexanol	1030	143.00	174.00	ND
2-Ethylcyclohexanol,c&t	1068	ND	ND	131.00

Table S1. (continued)				
2-Isopropyl-5-methyl-1-heptanol	1068	ND	ND	94.00
2-Octylcyclopropene-1-heptanol	2056	139.00	ND	ND
6,11-Dimethyl-2,6,10-dodecatrien-1-ol	1634	ND	ND	320.00
Bicyclo[4.1.0]heptan-3-ol, 4,7,7-trimethyl-, (1.alpha.,3.alpha.,4.alpha.,6.alpha.)-	1125	1409.00	ND	ND
Ethanol	463	ND	ND	58169.00
Ethanol, 2-(tetradecyloxy)-	1930	ND	1265.00	ND
Levomenthol	1164	ND	128.00	ND
Linalool	1082	20500.00	6853.00	4148.00
n-Tridecan-1-ol	1556	ND	ND	77.00
Phenylethyl Alcohol	1136	2158.00	9412.00	5138.00
Olefins				
(E)-.beta.-Famesene	1440	308.00	ND	ND
.alpha.-Pinene	948	199.00	ND	ND
.beta.-Bisabolene	1500	ND	200.00	ND
.beta.-Myrcene	958	6180.00	3406.00	ND
1,3,5,7-Cyclooctatetraene	888	92.00	ND	ND
1,3,6-Octatriene, 3,7-dimethyl-, (Z)-	976	621.00	ND	ND
1,3-Cyclohexadiene, 5-(1,5-dimethyl-4-hexenyl)-2-methyl-, [S-(R*,S*)]-	1451	299.00	409.00	239.00
1-Eicosene	1999	433.00	ND	ND
1-Hexene, 3,5,5-trimethyl-	757	171.00	ND	ND
1-Undecene, 8-methyl-	1140	ND	ND	425.00
2,4,6-Octatriene, 2,6-dimethyl-, (E,Z)-	993	181.00	ND	ND
Bicyclo[3.1.0]hex-2-ene, 2-methyl-5-(1-methylethyl)-	902	325.00	ND	ND
Bicyclo[4.1.0]-3-heptene, 2-isopropenyl-5-isopropyl-7,7-dimethyl-	1281	175.00	ND	ND
Caryophyllene	1494	3776.00	2969.00	ND
Caryophyllene oxide	1507	ND	143.00	ND
Copaene	1221	916.00	556.00	252.00
Cyclohexene, 3-(1,5-dimethyl-4-hexenyl)-6-methylene-, [S-(R*,S*)]-	1446	497.00	631.00	287.00
Cyclohexene, 4-ethenyl-4-methyl-3-(1-methylethenyl)-1-(1-methylethyl)-, (3R-trans)-	1377	578.00	341.00	168.00
D-Limonene	1018	62397.00	ND	ND
Germacrene D	1515	198.00	ND	ND
Humulene	1579	666.00	621.00	ND
Naphthalene, decahydro-4a-methyl-1-methylene-7-(1-methylethenyl)-, [4aR-(4a.alpha.,7.alpha.,8a.beta.)]-	1469	418.00	504.00	ND
Nonadecane, 9-methyl-	1945	ND	ND	256.00
Alkanes				
10-Methylnonadecane	1945	ND	ND	115.00
11-Methyltricosane	2343	726.00	ND	ND
2-Bromotetradecane	1645	ND	909.00	ND
2-Cyclohexylnonadecane	2506	ND	ND	193.00
2-Methylhexacosane	2641	1076.00	315.00	ND
2-Methyltetracosane	2442	794.00	333.00	94.00
3-Ethyl-3-methylheptane	931	ND	ND	223.00
5,5-Diethylpentadecane	1825	91.00	ND	ND
9-methylheptadecane	1746	311.00	ND	ND
Bicyclo[3.1.0]hexane, 4-methylene-1-(1-methylethyl)-	897	4335.00	1081.00	462.00
Bicyclo[5.2.0]nonane, 2-methylene-4,8,8-trimethyl-4-vinyl-	1407	1038.00	ND	ND
Cyclohexane, [6-cyclopentyl-3-(3-cyclopentylpropyl)hexyl]-	2592	656.00	ND	ND

Table S1. (continued)				
Cyclohexane, 1-ethenyl-1-methyl-2,4-bis(1-methylethenyl)-, [1S-(1.alpha.,2.beta.,4.beta.)]-	1398	367.00	335.00	ND
Cyclohexane, 1-methylene-4-(1-methylethenyl)-	1013	ND	189.00	143.00
Cyclopentane, nonyl-	1456	173.00	ND	ND
Cyclotetradecane	1679	ND	109.00	ND
Decane, 2,3,5,8-tetramethyl-	1156	ND	ND	72.00
Dodecane	1214	512.00	346.00	389.00
Dodecane, 2,6,10-trimethyl-	1320	412.00	217.00	ND
Dodecane, 2,6,11-trimethyl-	1320	551.00	185.00	ND
Dodecane, 2-methyl-	1249	ND	225.00	107.00
Dodecane, 3-cyclohexyl-	1810	201.00	ND	ND
Dodecyl nonyl ether	2185	85.00	ND	ND
Eicosane	2009	6818.00	3370.00	2364.00
Eicosane, 2,4-dimethyl-	2080	ND	ND	114.00
Heneicosane	2109	2758.00	2051.00	514.00
Heptadecane	1711	5600.00	6235.00	626.00
Heptadecane, 2-methyl-	1746	ND	ND	175.00
Heptadecane, 3-methyl-	1746	677.00	ND	173.00
Heptadecane, 4-methyl-	1746	388.00	ND	ND
Heptadecane, 7-methyl-	1746	682.00	390.00	75.00
Hexadecane	1612	ND	ND	136.00
Hexadecane, 1-iodo-	2026	459.00	350.00	ND
Hexadecane, 2,6,10,14-tetramethyl-	1753	590.00	ND	316.00
Hexadecane, 2,6,11,15-tetramethyl-	1753	ND	795.00	504.00
Hexane, 2,2-dimethyl-	732	ND	ND	150.00
n-Nonylcyclohexane	1576	470.00	492.00	ND
Nonane, 5-(2-methylpropyl)-	1185	ND	ND	245.00
Nonane, 5-butyl-	1249	816.00	ND	ND
Nonane, 5-methyl-5-propyl-	1229	ND	229.00	ND
Nonyl tetradecyl ether	2383	1755.00	1726.00	ND
Octacosane	2804	ND	298.00	ND
Octadecane	2036	ND	387.00	ND
Octadecane, 1-chloro-	2036	170.00	990.00	634.00
Octadecane, 3-methyl-	1846	964.00	ND	ND
Octadecane, 5-methyl-	1846	696.00	453.00	ND
Octane, 1,1'-oxybis-	1688	ND	109.00	ND
Octane, 2-methyl-	852	157.00	ND	ND
Octane, 4,5-dipropyl-	1285	366.00	330.00	ND
Oxirane, decyl-	1304	213.00	ND	ND
Oxirane, tetradecyl-	1702	ND	125.00	304.00
Pentadecane	1512	1102.00	ND	920.00
Pentadecane, 2,6,10,14-tetramethyl-	1653	3180.00	1242.00	397.00
Pentadecane, 4-methyl-	1548	432.00	693.00	126.00
Pentadecane, 7-methyl-	1548	145.00	ND	ND
Pentadecane, 8-hexyl-	2045	2730.00	1155.00	461.00
Pentane, 2,2,4-trimethyl-	668	ND	147.00	ND
Tetracosane	2407	ND	ND	135.00
Tetradecane, 1-iodo-	1827	240.00	ND	ND
Tetradecane, 5-methyl-	1448	420.00	ND	ND
Tetradecane, 6,9-dimethyl-	1483	ND	ND	125.00
Triacontane, 1-iodo-	3418	ND	349.00	ND

Table S1. (continued)

Tridecane, 3-methylene-	1380	311.00	ND	ND
Tridecane, 4-cyclohexyl-	1909	910.00	ND	ND
Nonadecane	1910	298.00	ND	ND
Other categories				
1,1'-Biphenyl, 2,2',5,5'-tetramethyl-	1820	904.00	702.00	ND
2-Methoxy-4-vinylphenol	1293	ND	179.00	183.00
Benzene, (2,2-dimethylpropyl)-	1107	ND	ND	122.00
Benzene, 1-(1,5-dimethyl-4-hexenyl)-4-methyl-	1524	473.00	911.00	262.00
Benzene, 1,2,4-trichloro-	1220	109.00	ND	ND
Benzene, 1,2-dichloro-	1040	750.00	750.00	750.00
Butylated Hydroxytoluene	1668	387.00	373.00	269.00
o-Isopropenyltoluene	1073	81.00	ND	ND
Phenol, 2,4,6-tri-tert-butyl-	1882	ND	ND	119.00
Phenol, 4-ethyl-	1114	ND	861.00	647.00
Toluene	1556	ND	ND	163.00
(+)-4-Carene	919	359.00	ND	ND
1,1'-Bicyclohexyl, 4,4'-dimethyl-	1463	ND	348.00	ND
1-Isopropyl-4,7-dimethyl-1,2,3,5,6,8a-hexahydronaphthalene	1469	350.00	362.00	ND
1-Octadecanesulphonyl chloride	2493	ND	347.00	120.00
1-Octadecyne	1808	142.00	ND	ND
2-Decenal, (E)-	1212	370.00	497.00	653.00
3-Carene	948	1270.00	509.00	333.00
Anethole	1190	750.00	279.00	434.00
Azulene	1490	ND	ND	206.00
Bicyclo[7.2.0]undec-4-ene, 4,11,11-trimethyl-8-methylene-	1494	799.00	ND	ND
Decyl octyl ether	1886	88.00	ND	ND
Diallyl disulphide	1099	97.00	ND	ND
Di-n-decylsulfone	2516	ND	ND	222.00
Furan, 2-pentyl-	1040	ND	ND	3637.00
Hydrazine, methyl-	1235	71429.00	18092.00	ND
Naphthalene	1231	221.00	215.00	ND
Naphthalene, 1,2-dihydro-1,1,6-trimethyl-	1396	ND	ND	118.00
Pyrene, hexadecahydro-	1502	948.00	ND	ND
Pyridine, 3,5-dichloro-	1034	48188.00	50468.00	47970.00
trans-.beta.-Ocimene	976	ND	164.00	ND
